# Establishment of skin cutaneous melanoma prognosis model based on vascular mimicry risk score

**DOI:** 10.1097/MD.0000000000036679

**Published:** 2024-02-16

**Authors:** Yubo Wang, Linxuan Zou, Mingzhi Song, Junwei Zong, Shouyu Wang, Lei Meng, Zhuqiang Jia, Lin Zhao, Xin Han, Ming Lu

**Affiliations:** a Dalian Medical University, Dalian, China; b Department of Trauma and Tissue Repair Surgery, Dalian Municipal Central Hospital, Dalian, China; c Department of Orthopaedic Surgery, The First Affiliated Hospital of Dalian Medical University, Dalian, China; d The First Affiliated Hospital of Nanhua Medical University, Hengyang, China; e The First Affiliated Hospital of Dalian Medical University, Dalian, China; f Naqu People’s Hospital, Tibet, China; g Department of Quality Management, Dalian Municipal Central Hospital, Dalian, China; h Department of Orthopaedic Surgery, The Second Affiliated Hospital of Dalian Medical University, Dalian, China; i Department of Trauma and Tissue Repair Surgery, Dalian Municipal Central Hospital of Dalian Medical University, Dalian, China.

**Keywords:** prognostic, skin cutaneous melanoma, TCGA, vasculogenic mimicry

## Abstract

Studies have indicated that Vascular mimicry (VM) could contribute to the unfavorable prognosis of skin cutaneous melanoma (SKCM). Thus, the objective of this study was to identify therapeutic targets associated with VM in SKCM and develop a novel prognostic model. Gene expression data from The Cancer Genome Atlas (TCGA) and Genotype-Tissue Expression (GTEx) were utilized to identify differentially expressed genes (DEGs). By intersecting these DEGs with VM genes, we acquired VM-related DEGs specific to SKCM, and then identified prognostic-related VM genes. A VM risk score system was established based on these prognosis-associated VM genes, and patients were then categorized into high- and low-score groups using the median score. Subsequently, differences in clinical characteristics, gene set enrichment analysis (GSEA), and other analyses were further presented between the 2 groups of patients. Finally, a novel prognostic model for SKCM was established using the VM score and clinical characteristics. 26 VM-related DEGs were identified in SKCM, among the identified DEGs associated with VM in SKCM, 5 genes were found to be prognostic-related. The VM risk score system, comprised of these genes, is an independent prognostic risk factor. There were significant differences between the 2 patient groups in terms of age, pathological stage, and T stage. VM risk scores are associated with epithelial biological processes, angiogenesis, regulation of the SKCM immune microenvironment, and sensitivity to targeted drugs. The novel prognostic model demonstrates excellent predictive ability. Our study identified VM-related prognostic markers and therapeutic targets for SKCM, providing novel insights for clinical diagnosis and treatment.

## 1. Introduction

Skin cutaneous melanoma (SKCM) is a malignant tumor that arises from melanocytes located in the human skin. It is characterized by its high degree of malignancy and aggressiveness. SKCM represents the most prevalent form of skin cancer, exhibiting the highest mortality rate, and its incidence is increasing.^[1–3]^ Due to often inconspicuous early clinical manifestations, patients may experience delayed treatment initiation. Elevated rates of invasion and metastasis constitute significant factors contributing to the unfavorable prognosis of SKCM. Currently, the standard treatment for SKCM involves surgical excision of the primary lesion in the absence of metastasis.^[4]^ Upon occurrence of metastasis, SKCM exhibits high resistance to conventional chemotherapy and radiotherapy interventions.^[5]^ Therefore, to address this situation, it is crucial to delve deeper into the occurrence, development, and metastasis mechanism of SKCM, identify novel therapeutic targets associated with survival, and enhance the methods used for evaluating patient prognosis in order to guide clinical treatment effectively.

Vascularization is a crucial aspect and necessary process in the development of SKCM.^[3]^ Research has demonstrated that when the diameter exceeds than 2 mm, sufficient blood supply is necessary to sustain viable tumor tissue.^[3]^ The conventional mode of angiogenesis involving endothelial cell germination and proliferation serves as the primary mechanism for blood supply.^[6]^ Consequently, anti-angiogenic drugs have been developed and employed for tumor-specific therapy, inducing tumor tissue ischemia and hypoxia and inhibit tumor development.^[7]^ Nevertheless, the efficacy of the presently available drugs for malignant melanomas remains unsatisfactory.

A tubular structure of human melanoma was described by Maniotis et al in 1999. The wall of the tubular structure was surrounded by a basement membrane stained with PAS (+) and CD31 (−), and lined with tubular structures of tumor cells. Subsequent studies revealed the tube capability to blood supply to tumor tissue and termed it “vascular mimicry (VM).”^[8]^ Despite the debated biological function of this structure as a tumor blood supply, subsequent studies have led to an enhanced comprehension of its nature. Abundant research has demonstrated the presence and linkage of VM with unfavorable prognosis in cancers, including sarcoma, breast cancer, lung cancer, glioma, and other malignant tumors. This mode of tumor blood supply, which differs from traditional vascular endothelial angiogenesis, offers a novel approach for targeted tumor therapy.^[9–12]^ In recent years, some key pathways and related mechanisms of VM have been reported, among which VE-cadherin and EphA2 are the first 2 proteins identified for VM formation, playing a crucial role in the vascular signaling pathways that contribute to VM.^[13]^ In melanoma, malignant melanoma initiation cells contribute to VM formation through the expression of ATP-binding cassette (ABC) member ABCB5. This is primarily mediated through the VEGFR1-mediated signal transduction pathway.^[14]^ Hypoxia is also considered a critical pathway for VM, and the MEK/ERK pathway plays a role in VM formation in certain malignancies under hypoxic conditions.^[15]^ Furthermore, various embryonic stem cell-related genes, such as OCT4 and SOX2, are upregulated in lung cancer and associated with the development of VM.^[16]^ Due to the significant influence of VM on the occurrence and development of tumor diseases, treatment methods, and patient prognosis, it is clinically imperative to conduct comprehensive research at the genetic level to elucidate the underlying mechanisms of VM-related genes, establish the correlation between VM and SKCM patient prognosis, and identify potential therapeutic targets related to VM. Nevertheless, there is a scarcity of studies concerning SKCM in this area. Thus, in this study, we utilized biological information databases such as TCGA,CTEx and Gene Expression Omnibus (GEO) to identify VM genes obtained from the Genecards database. Subsequently, we established a prognostic VM risk score system. Utilizing this system, a comprehensive analysis encompassing gene function enrichment, tumor drug sensitivity, immune infiltration, and other directions was performed. Additionally, a novel prognostic model for SKCM was established using these findings. This study is expected to contribute to the treatment of patients with SKCM.

## 2. Methods

### 2.1. VM genes, expression data of SKCM and normal human skin, clinical information of SKCM

We downloaded VM genes from Genecards (http://www.genecards.org). Next, we obtained the expression matrix and clinical information of SKCM from The Cancer Genome Atlas (TCGA, http://portal.gdc.cancer.gov/). The dataset GSE19234^[17]^ from the GEO (https://www.ncbi.nlm.nih.gov/geo) was downloaded for external validation. Next, we downloaded the normal human skin expression matrix from Genotype-Tissue Expression (GTEx) database of the UCSC Xena database (http://xena.ucsc.edu/) as the control group. The downloaded gene expression data of TCGA and GTEx were merged and the batch effect is removed by“sva”R package. For the clinical data, we removed missing and incomplete information.

### 2.2. The identification of differentially expressed VM genes

Under the standards of | logFC | > 1 and false discovery rate (FDR) < 0.05, we confirmed SKCM differentially expressed genes (DEGs) using the R language, “limma” package. We performed an intersection analysis of these genes with VM genes obtained from the Genecards website to identify DEGs associated with VM in SKCM., a Venn diagram was generated using the “VennDiagram” R package to visualize the overlap between upregulated and downregulated genes in SKCM and VM DEGs in SKCM. Heatmaps of DEGs were made by using “heatmap” R package and a labeled volcano map was made by using R package “ggplot2” and “ggrepel.” to show VM DEGs of SKCM.

### 2.3. Gene ontology (GO) and KEGG functional enrichment analysis

GO analysis, including molecular function (MF), biological processes (BP) and cell components (CC), as well as Kyoto Encyclopedia of Genes and Genomes (KEGG) pathway analysis was presented through the “ClusterProfler.” FDR and *P* < .05 were standards of significantly enriched.^[18]^

### 2.4. The construction of VM prognostic risk score system

After removing the missing values, we used overall survival data of TCGA clinical information as a prognostic indicator. Univariate COX regression to preliminatively evaluate the correlation between VM-related SKCM differential expression genes and prognosis by “survival” R package, and the least absolute shrinkage and selection operator (LASSO) algorithm was used to screen out VM DEGs that were associated with the overall survival of SKCM patients by using the “glmnet” R package.^[19]^ Finally, multivariate Cox regression analysis was used to identify VM related genes associated with overall survival in SCKM patients and construct VM prognostic risk score system of SKCM. The model was expressed as follows: VM risk score = 


$$\begin{array}{l} \sum_{i=1}^{n}coef_{i}^{*}mRNA_{i} \end{array}$$

“Coef” represents the COX regression coefficient of a gene, and “mRNA” represents the gene expression. Patients of SCKM were divided into high and low risk groups based on the median VM risk score. To test the accuracy of the scoring system, it was verified by bootstrapping validation (1000 bootstrap resamples) and the GEO dataset GSE19234. Results are presented as the Kaplan–Meier survival curve and the time-independent receiver operating characteristic (ROC) curve for 1, 3, and 5 years overall survival. Furthermore, we analyzed the differences in the clinical information between the 2 groups.

### 2.5. Gene set enrichment analysis (GSEA)

In order to investigate the biological characteristics and pathway enrichment of genes involved in VM risk scoring system deeply, the gene sets of KEGG and GO were downloaded in.gmt file format from the Molecular Signatures Database (MSigDB) of GSEA (https://www.gsea-msigdb.org/gsea/index.jsp),^[20]^ the analysis was present by “ClusterProfler” and “enrichplot” R package, standards of significantly enriched were same as GO and KEGG analysis above FDR and *P* < .05 were standards of significantly enriched.

### 2.6. Evaluation of SKCM immune microenvironment

The correlation between VM genes and the infiltration level of several types of immune cells was evaluated using CIBERSORT R script v1.03. Next, the CIBERSORT and single-sample gene set enrichment analysis algorithms were used to evaluate the differences in the immune cell microenvironment between the VM high and low risk group by using the “GSVA” R packages. The expression of several immune checkpoints was further studied to identify immunotherapeutic targets. Statistical significance was set at *P* < .05.

### 2.7. Searching for sensitive drugs

Based on the Genomics of Drug Sensitivity in Cancer (https://www.cancerrxgene.org/), we used the “oncoPredict” R package to predict drug sensitivity.^[21]^ Half-maximal inhibitory concentration (IC50) could indicate the corresponding concentration of an antitumor drug when the ratio of inducing apoptosis of tumor cells was equal to 50%, and was used as a monitoring index for the sensitivity of antitumor drugs. Combined with the VM scores of all patients, the drug sensitivity of the 2 groups was analyzed and presented as a boxplot. Statistical significance was set at *P* < .05.

### 2.8. Screening of SKCM independent prognostic risk factors and Nomogram establishment of SKCM prognostic model

We performed univariate COX regression for VM risk scores and other clinical information, such as age, gender, pathological stage, T stage, N stage, M stage. The LASSO algorithm and multivariate Cox regression analysis were used to determine the independent prognostic risk factors of SKCM. Next, a prognostic model for SKCM was established using the VM risk score and clinical information, it was presented as a nomogram to assess 1, 3, and 5 years overall survival of SKCM patients. The ROC and calibration curves were used to evaluate the accuracy of the prognostic model.

### 2.9. Statistical method

Statistical analysis of all data was performed using Version 4.2.1 R software, Wilcoxon rank sum test was used for analyzing between-group variation of quantitative variables, Fisher precision probability test was used for analyzing between-group variation of qualitative variables, Kaplan–Meier survival curves were analyzed using the log-rank test, and *P* < .05 were s standards of significant.

## 3. Results

### 3.1. Identification and enrichment analysis of VM DEGs of SKCM

**Figure 1.**
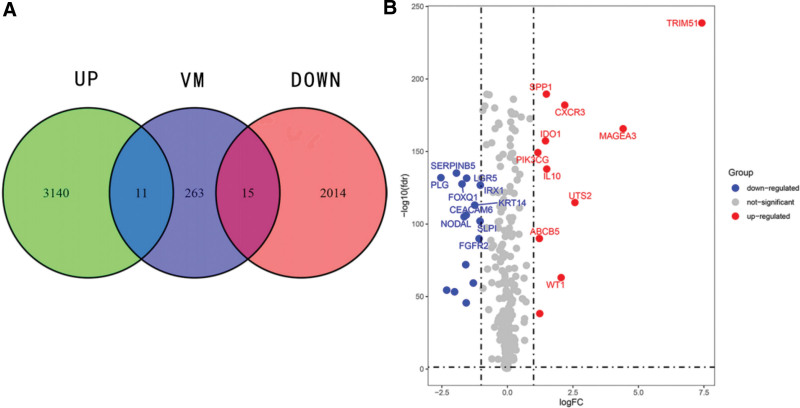
The identification of VM-related DEGs of SKCM. (A) Venn diagram, (B) volcano map showed VM-related DEGs of SKCM. DEGs = differentially expressed genes, VM = vascular mimicry.

**Figure 2.**
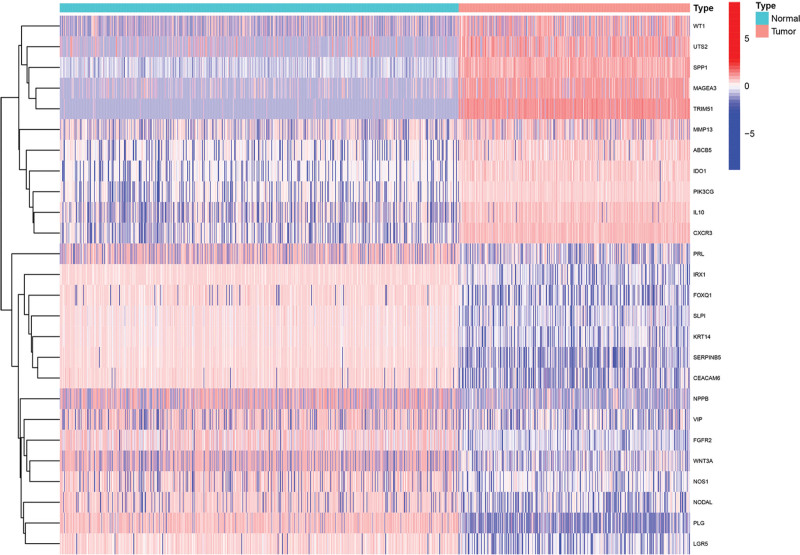
Heatmap showed VM DEGs of SKCM. DEGs = differentially expressed genes, SKCM = skin cutaneous melanoma, VM = vascular mimicry.

**Figure 3.**
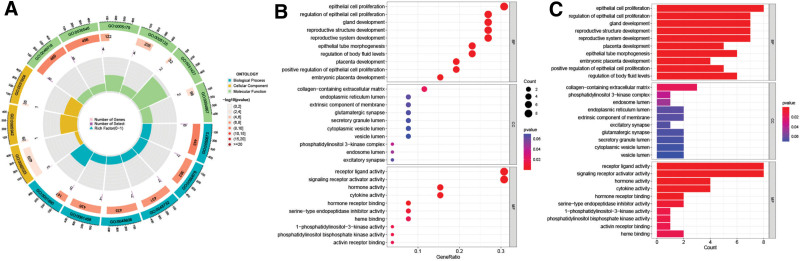
GO analysis of SKCM VM DEGs. (A) Circlize, (B) bubble, (C) barplot. DEGs = differentially expressed genes, GO = gene ontology, SKCM = skin cutaneous melanoma, VM = vascular mimicry.

**Figure 4.**
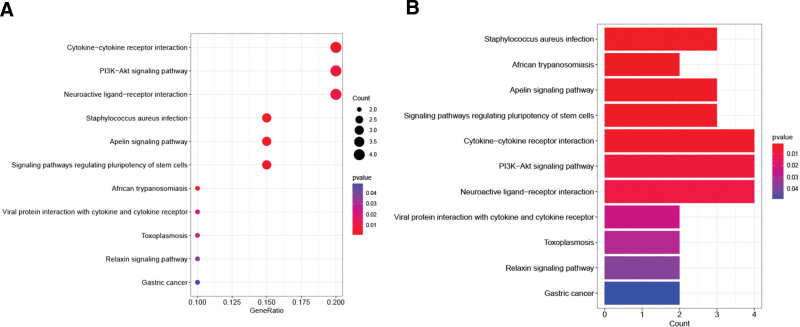
KEGG analysis of SKCM VM DEGs. (A) Bubble, (B) barplot. DEGs = differentially expressed genes, KEGG = Kyoto encyclopedia of genes and genomes, SKCM = skin cutaneous melanoma, VM = vascular mimicry.

A total of 5180 DEGs were identified from 471 tumor samples from TCGA and 812 normal samples from GTEx (Supplementary table S1, S2, http://links.lww.com/MD/L560, http://links.lww.com/MD/L562). These genes intersected 289 VM genes (Supplementary table S3, http://links.lww.com/MD/L563) downloaded from the Genecards database to obtain 26 VM DEGs of SKCM (Figs. 1 and 2, Supplementary table S4, http://links.lww.com/MD/L564), including 11 upregulated genes (WT1, UST2, SPP1, MAGEA3, TRIM51, MMP13, ABCB5, IDO1, PIK3CG, IL10, and CXCR3) and 15 downregulated genes (PRL, IRX1, FOXQ1, SLPI, KRT14, SERPINB5, CEACAM6, NPPB, VIP, FGFR2, WNT3A, NOS1, NODAL, PLG, and LGR5). The mainly enriched biological process (BP) terms of SKCM VM-related DEGs were”(GO:0050673) epithelial cell proliferation,””(GO:0050678) regulation of epithelial cell proliferation,””(GO:0048732) gland development,””(GO:0048608) reproductive structure development,””(GO:0061458) reproductive system development,””(GO:0001890) placenta development,” and”(GO:0060562) epithelial tube morphogenesis,” the mainly enriched cellular component (CC) terms were “(GO:0062023) collagen-containing extracellular matrix,””(GO:0005942) phosphatidylinositol 3-kinase complex,” and”(GO:0031904) endosome lumen,” the mainly enriched molecular function(MF) terms were”(GO:0048018) receptor ligand activity,””(GO:0030546) signaling receptor activator activity,””(GO:0005179) hormone activity,””(GO:0005125) cytokine activity,””(GO:0051427) hormone receptor binding,” and”(GO:0004867) serine-type endopeptidase inhibitor activity” (Fig. 3). SKCM VM-related DEGs were mainly enriched in the PI3K-Akt signaling pathway, neuroactive ligand-receptor interaction, cytokine-cytokine receptor interaction, apelin signaling pathway, and Staphylococcus aureus infection (Fig. 4).

### 3.2. Five VM genes associated with SKCM prognosis were finally identified

**Figure 5.**
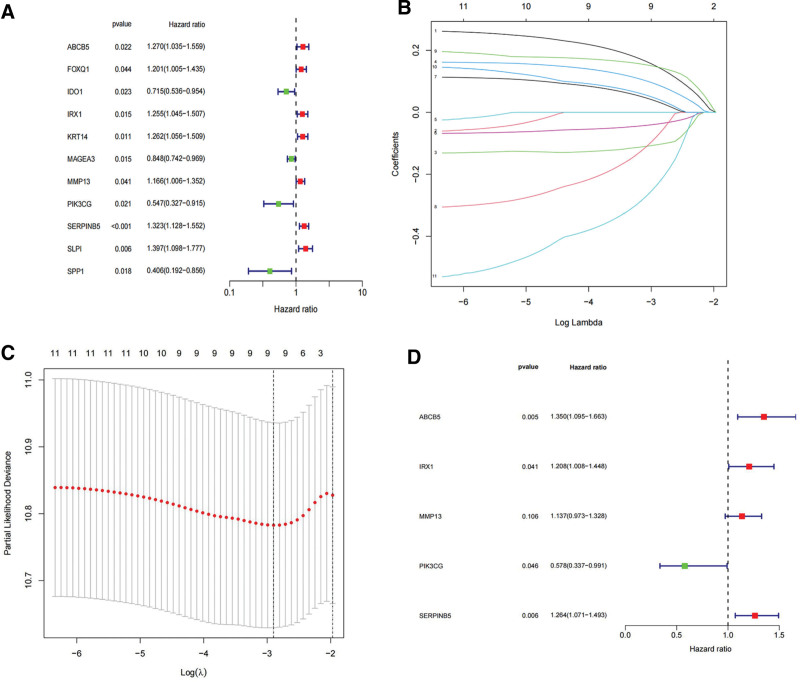
Identification of prognosis - related VM DEGs. (A) Prognosis - related VM DEGs of SKCM were identified by Univariate COX regression, (B-C) LASSO, (D) multivariate Cox regression. DEGs = differentially expressed genes, LASSO = least absolute shrinkage and selection operator, SKCM = skin cutaneous melanoma, VM = vascular mimicry.

Eleven VM-related SKCM DEGs, namely ABCB5, FOXQ1, IDO1, IRX1, KRT14, MAGEA3, MMP13, PIK3CG, SERPINB5, SLPI, and SPP1, were associated with prognosis. Based on these results, we screened 9 prognostic-related genes using the LASSO algorithm. Subsequent to performing multivariate Cox regression analysis, we identified 5 genes (ABCB5, IRX1, MMP13, PIK3CG, and SERPINB5) for the establishment of a prognostic risk score system associated with VM (Figs. 5A–D).

### 3.3. Establishment and verification of risk score system

**Figure 6.**
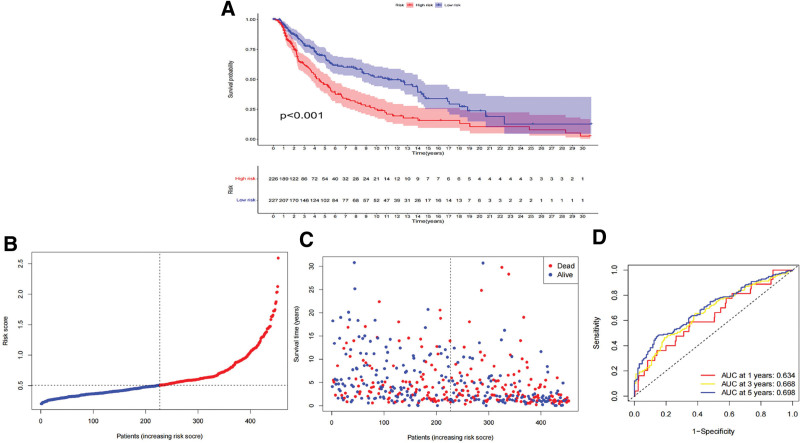
Measurement and prediction accuracy of VM risk score system. (A–C) Survival analysis between high and low VM risk score group based on TCGA set, (D) Time-independent ROC analysis shown prediction accuracy of overall survival based on TCGA set. ROC = receiver operating characteristic, TCGA = The Cancer Genome Atlas, VM = vascular mimicry.

**Figure 7.**
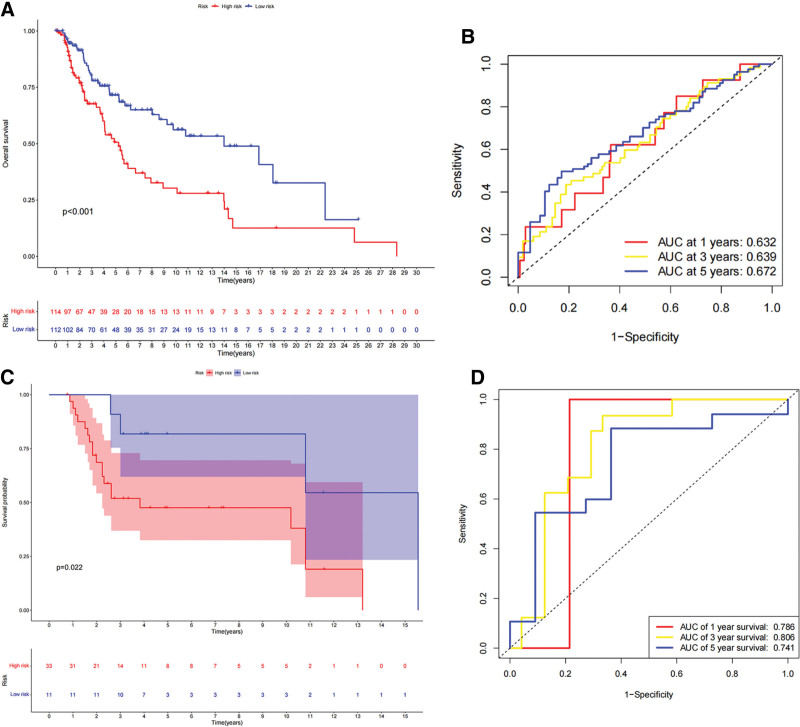
Measurement and prediction accuracy of VM risk score system. (A) Survival analysis between high and low VM risk score group based on bootstrapping validation, (B) Time-independent ROC analysis shown prediction accuracy of overall survival based on bootstrapping validation, (C) Survival analysis between high and low VM risk score group based on GSE19234, (D) Time-independent ROC analysis shown prediction accuracy of overall survival based on GSE19234. ROC = receiver operating characteristic, VM = vascular mimicry.

The COX regression coefficient (Supplementary table S5, http://links.lww.com/MD/L565) and expression level of each gene were used to construct a prognostic risk score system associated with VM. The VM risk score was calculated using the following formula: VM risk score = ABCB5 expression × 0.2998 + IRX1 expression × 0.18916 + MMP13 expression × 0.128263 + PIK3CG expression × (−0.54818) + SERPINB5 expression × 0.2344. Subsequently, based on the median value of all samples, the subjects were divided into high- and low-score groups (Supplementary table S6, http://links.lww.com/MD/L567). In the TCGA dataset, bootstrapping validation, or GEO dataset (Supplementary table S8, S9, http://links.lww.com/MD/L571, http://links.lww.com/MD/L573), survival analysis results (Figs. 6A, 7A, 7C) presented that the overall survival of patients in the low-score group was significantly better (*P* < .001). Riskline and riskpoint graphs (Fig. 6B and C) show that the VM score of patients was closely related to overall survival. The ROC curve (Fig. 6D) showed that this scoring system had a certain measurement and prediction accuracy, and the ROC curve after bootstrapping validation showed that the AUC values of the VM risk score model at 1-, 3-, and 5-years was 0.632, 0.639, 0.672, and 0.786 at 1-, 0.806 at 3- and 0.741 at 5-years in the GEO dataset verification, respectively, with good predictive performance (Fig. 7B and D).

### 3.4. VM risk score is closely related to other clinical information

**Figure 8.**
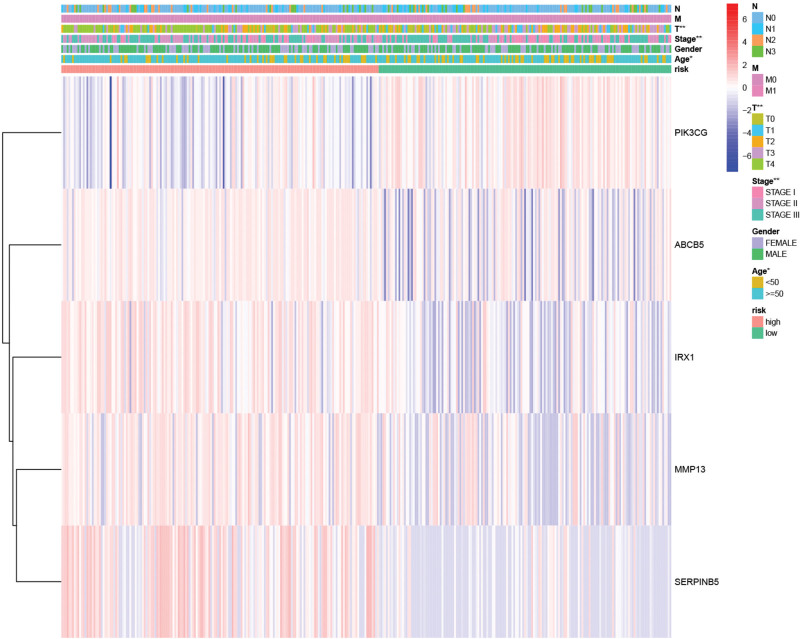
The difference of SKCM patients clinical information between VM high and low risk score group. * represents a significant difference, *P* < .05, ** represents a significant difference, *P < *.01. SKCM = skin cutaneous melanoma, VM = vascular mimicry.

**Figure 9.**
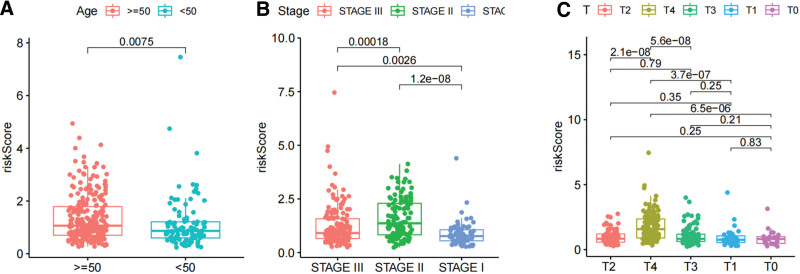
Differences in VM risk score between clinical subgroups. VM = vascular mimicry.

**Figure 10.**
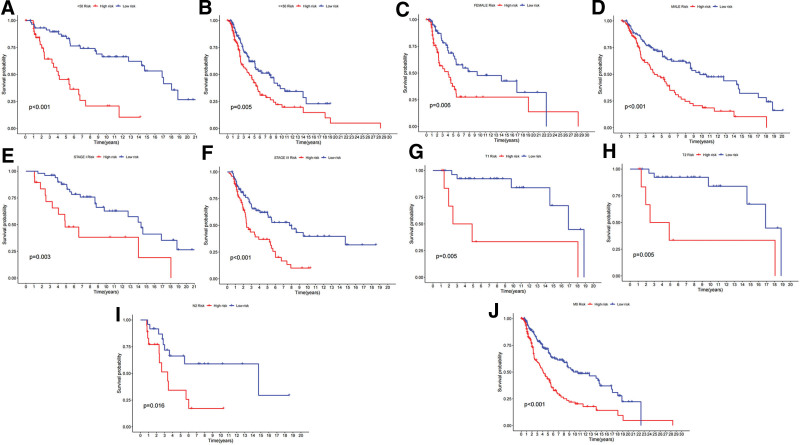
Stratified survival analysis of high-risk score and low-risk score group. (A–B) age, (C–D) gender, (E–F) pathological stage I and III, (G–H) T1 and T2 stage, (I) N2 stage, (J) M0 stage.

The Wilcoxon rank sum test and Fisher precision probability test revealed significant differences in the clinical information of SKCM patients between the 2 groups (Figs. 8 and 9, Supplementary table S7, http://links.lww.com/MD/L569), these differences encompassed pathological stage (*P* < .01), age (*P* = .0489), and T stage (*P* < .01). Notably, we found that this score model demonstrated favorable performance in predicting survival outcomes for specific patients subgroups categorized by characteristics such as age > 50 and < 50 years, male and female, pathological stage1, pathological stage 3, T1, T2, N2, and M0 (Fig. 10).

### 3.5. VM risk score is an independent prognostic risk factor

**Figure 11.**
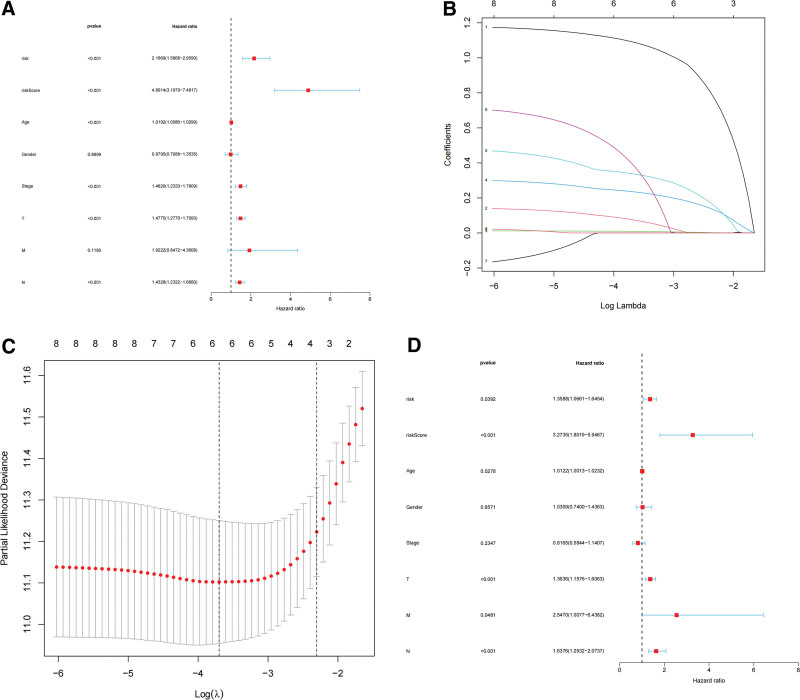
Identification of independent prognostic risk factor. (A) Univariate COX regression, (B–C) LASSO, (D) multivariate Cox regression. LASSO = least absolute shrinkage and selection operator.

Independent prognostic risk factors for SKCM were identified using a method previously used to identify prognostic genes associated with VM. The final results showed that independent prognostic factors included the VM risk score, age, and TNM stage of the tumor (Fig. 11A–D).

### 3.6. GSEA based on VM risk score

**Figure 12.**
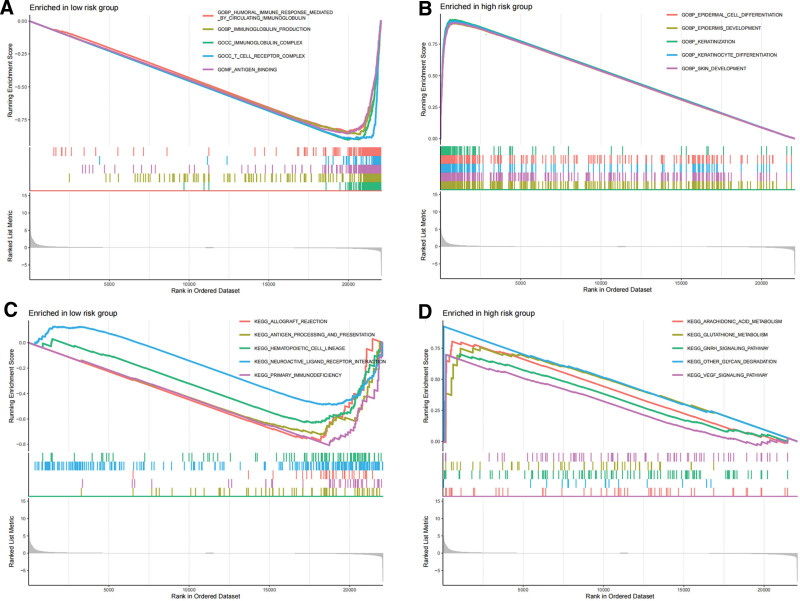
GSEA analysis. GSEA = gene set enrichment analysis.

The results of GSEA based on the gene expression in all samples and the high-low risk grouping of the samples (Supplementary table S1,S6, http://links.lww.com/MD/L560, http://links.lww.com/MD/L567) further explained the potential molecular mechanism of genes related to VM risk score, among which genes in the high score group were mainly enriched in biological process such as “epidermal cell differentiation,” “keratinization,” “keratinocyte differentiation.” The main enrichment pathways included the “VEGF signaling pathway,” “glutathione metabolism,” “arachidonic acid metabolism,” and “other glycan degradation pathways.” The enrichment results of the low-score group were associated with antibodies, immunoglobulins, and human immune functions (Fig. 12A–D).

### 3.7. Immune microenvironment analysis based on VM risk score

**Figure 13.**
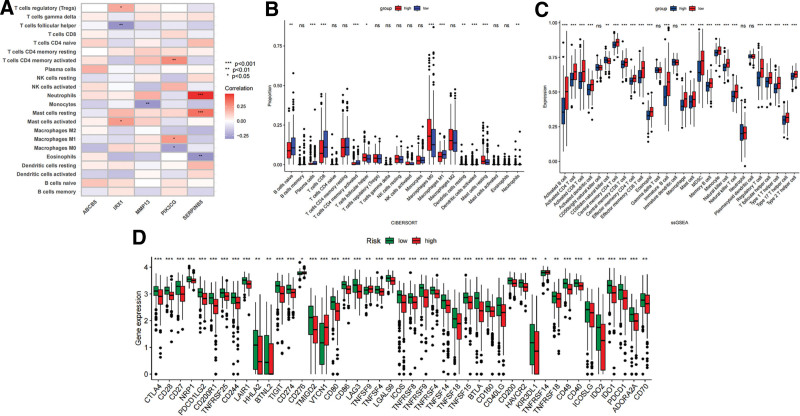
Analyses of tumor immune microenvironments. (A) Association of VM genes and immune cell infiltration. (B–C) Difference in immune cells infiltration levels between high and low VM score groups. (D) Expression of immune checkpoint between high and low VM score group. VM = vascular mimicry.

Among the 5 genes in the VM risk score system, the expression of IRX1 was positively correlated with the infiltration levels of activated mast cells and regulatory T cells (Tregs), and negatively correlated with follicular helper T cells. MMP13 showed a negative correlation with Monocytes, PIK3CG had a negative correlation with M1 Macrophages and activated memory CD4 T cells, as well as a negative correlation with M0 Macrophages. SERPINB5 exhibited a positive correlation with resting mast cells and neutrophils while showing a negative correlation with eosinophils (Fig. 13A).

The utilization of the CIBERSORT algorithm revealed differences in immune infiltration levels between the 2 groups. Specifically, the low VM score group exhibited significantly higher proportions of CD8 + T cells, activated CD4 + memory T cells, and M1 Macrophages. Conversely, some other types of cells, such as M0 Macrophages, demonstrated contrasting results. Further single sample gene set enrichment analysis results indicated that the proportion of most the vast majority of immune cell species was significantly higher in patients with low VM scores, suggesting an elevated immune status in this group. Based on the above results, we hypothesize a close association between the VM score system and the level of tumor immunity in patients with SKCM. Additionally, the analysis of VM score and immune checkpoints revealed a significant increase in the expression level of VTCN1 among patients in the high VM score group. Consequently, it is plausible that VM could contribute to invasion and metastasis of SKCM through the modulation of immune surveillance (Fig. 13B–D).

### 3.8. Drug sensitivity analysis and comparison

**Figure 14.**
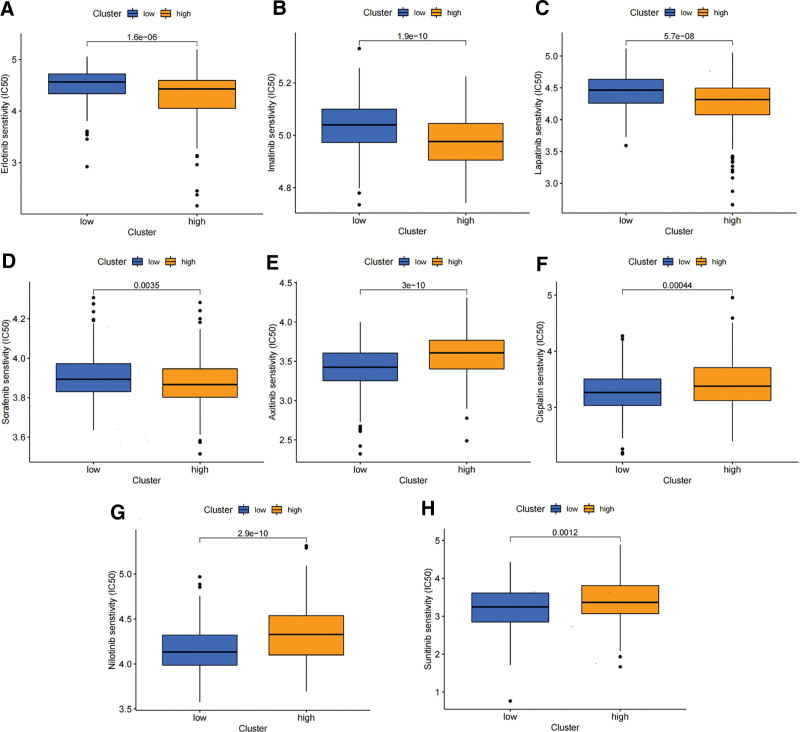
Drug sensitivity analysis.

Besides analyzing the level of immune infiltration, we conducted a comparison of tumor chemotherapy drug sensitivity between patients with high and low VM scores. The IC50 value served as the indicator for evaluating sensitivity. The IC50 values of erlotinib, imatinib, lapatinib, and sorafenib were significantly lower in the high-score group (all *P* < .001) (Fig. 14A–D), indicating that patients in this group may benefit from these drugs. Axitinib, cisplatin, nilotinib, and sunitinib levels were significantly lower in the low-score group (all *P* < .001) (Fig. 14E–H) indicating that patients in this group may benefit from these drugs.

### 3.9. Establishment of a SKCM prognostic model

**Figure 15.**
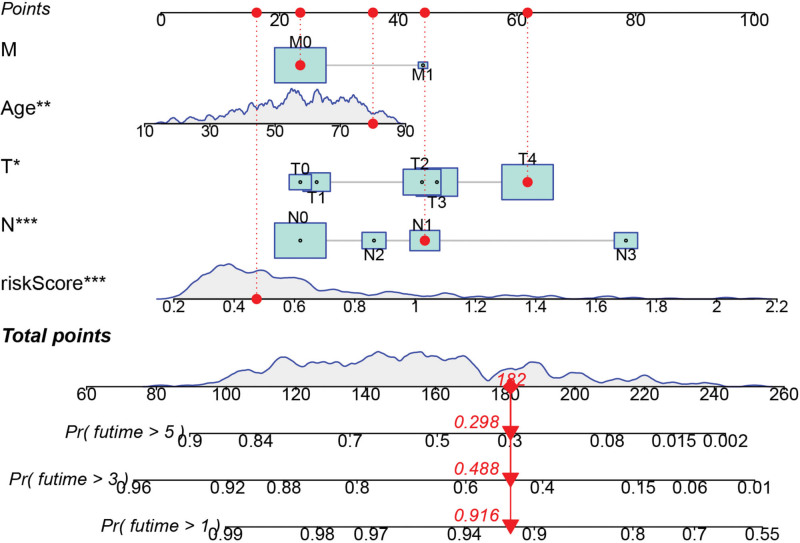
SKCM prognostic Nomogram of 1, 3, and 5-yr overall survival. SKCM = skin cutaneous melanoma.

**Figure 16.**
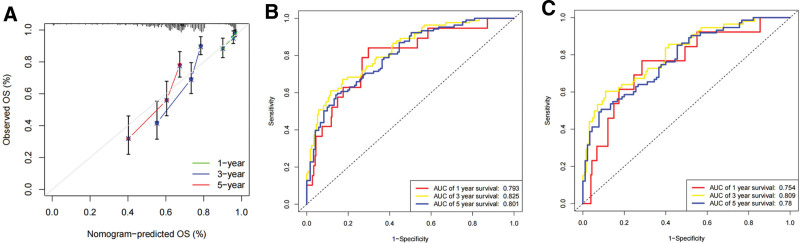
Evaluation of SKCM prognostic model prediction ability. (A) calibration curves. (B) Time-independent ROC analysis of SKCM prognostic model predicting the overall survival based on TCGA set. (C) Time-independent ROC analysis of SKCM prognostic model predicting the overall survival based on bootstrapping validation. ROC = receiver operating characteristic, SKCM = skin cutaneous melanoma, TCGA = The Cancer Genome Atlas.

**Figure 17.**
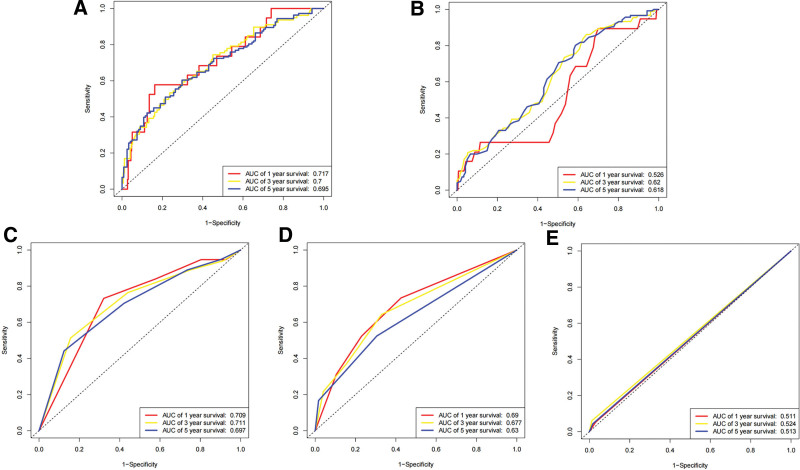
The ROC analysis of each risk factor. Prediction ability of prognostic model is better than the ability of (A) risk score, (B) age, (C) T stage, (D) N stage, (E) M stage. ROC = receiver operating characteristic.

This study employed a nomogram to demonstrate a prognostic model comprising independent prognostic risk factors for SKCM (Fig. 15). We validated the consistency between predicted and actual survival rates. (Fig. 16A), and the prognostic model exhibited remarkable predictive performance for 1, 3, and 5-years overall survival of SKCM patients, as evidenced by AUC values of 0.793, 0.825, and 0.801, respectively. Bootstrapping validation further supported the superior performance of our model, with AUC results of 0.764, 0.809 and 0.78 at 1-, 3-, and 5-years, respectively (Fig. 16B–C). Notably, the model outperformed individual risk factors alone. highlighting its utility as an ideal tool for prognostic estimation of SKCM (Fig. 17A–E).

## 4. Discussion

Despite representing a small proportion of malignant skin tumors, SKCM exhibits highest mortality rate compared to other skin malignancies.^[22]^ Its incidence continues to rise annually, making it one of the fastest growing forms of malignant tumors.^[23]^ Due to its highly malignant and aggressive nature, treatment options are limited, leading to a poor prognosis, as metastasis to distant and visceral organs is nearly incurable.^[24]^ In recent years, as research on SKCM treatment has progressed, there has been a noticeable decrease in the mortality rate among SKCM patients. However, SKCM remains a significant public health concern that warrants attention. Therefore, investigating therapeutic targets and establishing accurate prognostic methods are helpful in designing personalized treatment plans and enhancing therapeutic outcomes.^[25]^ Therapeutic resistance is currently generated during the treatment of metastatic melanomas. Although factors such as DNA repair ability, drug delivery, cell proliferation, angiogenesis, and apoptotic resistance are known factors contribute to drug resistance, the insensitivity of melanoma to chemotherapy regimens still lacks a clear explanation. The most plausible hypothesis for this phenomenon is that melanoma may possess additional resistance mechanism and the discovery of VM could offer a novel avenue to address this question.

VM represents a vascular endothelial-independent mechanism of tumor blood supply, and research on its mechanism of formation is still advancing. Previous studies have shown that the microenvironment, epithelial-mesenchymal transition (EMT), and cancer stem cells are closely related to VM formation. Under the hypoxic conditions, cancer stem cells adopt endothelial cell characteristics while losing their epithelial cell features through epithelial mesenchymal transition, enabling them to acquire vital oxygen and nutrients crucial for growth. Consequently, morphological changes lead to the formation of VM conduits that establish connections with the vascular microcirculation network.^[26,27]^ For instance, ZEB 1 expedites VM formation through EMT. VM has been implicated in the development of resistance to anti-angiogenic drugs. When the vascular endothelial cell pathway is blocked, VM can still provide a blood supply to the tumor tissue, and the hypoxic microenvironment caused by anti-angiogenic drugs promotes VM formation.^[28,29]^ Since VM structures consist entirely of tumor cells, their formation leads to direct exposure of tumor cells to the bloodstream, significantly elevating the risk of metastasis and adversely impacting prognosis.^[30]^ Therefore, identifying VM-related therapeutic targets that influence the prognosis of SKCM and establishing prognostic models carry clinical significance.

In this study, to present a prognostic-related VM risk-scoring system, we identified 5 VM-related genes associated with prognosis, including ABCB5, IRX1, MMP13, PIK3CG, and SERPINB5. The scoring system offers a potential and viable approach for assessing the clinical prognosis of SKCM. ABCB5 is predominantly expressed in melanocytes and studies have indicated its involvement in tumor progression and resistance to various drugs in malignant tumors, including melanoma.^[31,32]^ It affects the proliferative activity of melanoma cells and participates in VM.^[33]^ Additionally, ABCB5 has been shown to promote tumor invasion by regulating EMT-related transcription factors.^[29]^ IRX1 is one of the 6 members of the Iroquois homeobox gene family. Research indicated that IRX1 plays a significant role in suppressing peritoneal diffusion and lung metastasis in gastric cancer by exerting anti-angiogenesis and anti-VM effects.^[34]^ Moreover, hypomethylation of IRX1 may elevate the risk of lung metastasis.^[35]^ There are few reports on the relationship between this gene and SKCM cell proliferation and invasion, angiogenesis, and VM, necessitating further studies. During SKCM metastasis, basement membrane degradation and extracellular matrix remodeling occur, this process is mediated by proteolytic enzymes such as matrix metalloproteinases (MMPs), a high level of MMPs expression is one of the most important prerequisites for VM formation.^[36]^ MMP-13, a member of the MMP gene family, has been implicated in the invasion and progression of SKCM.^[37]^ Zigrino et al observed that inoculation of mouse melanoma B16F1 cells in the derma resulted in significant inhibition of tumor growth and distant metastasis upon knockout of MMP-13, particularly at early time points. These findings suggest that MMP-13 promotes the growth and organ-specific metastasis of SKCM.^[38]^ In this study, enrichment analysis of the KEGG pathway revealed a close relationship between the PI3K-Akt pathway and SKCM. As a positive regulator of this signaling axis, PI3K serves as a crucial signaling hub for signal propagation, cell activation, polarization, and morphological adaptation. Moreover, PI3KCG (PI3K-γ) is a potential therapeutic target for various human diseases.^[39]^ Studies have shown that PIK3CG expression levels are correlated with patient survival. A multi-omics study of melanoma brain metastases revealed a potential association between PIK3CG and multiple brain metastases.^[40]^ Furthermore, other studies have shown that the inhibition of PI3KCG can suppress tumor cell growth, reverse epithelial mesenchymal transformation, and reduce tumor metastasis in vivo. However, the therapeutic effect on tumor cell proliferation is considerably superior to its impact on primary tumor growth. These findings suggest that targeting PI3KCG is more suitable for the treatment and prevention of tumor spread and metastasis.^[41]^ SERPINB5 belongs to the serpentine superfamily and belongs to the ov-serpin subfamily. SERPINB5 inhibits tumor-induced angiogenesis, invasion, and metastasis through direct binding to extracellular matrix components. Given the presence of extracellular matrix remodeling during VM, SERPINB5 may inhibit VM formation through directly binding to extracellular matrix. Reduced SERPINB5 expression has been associated with the progression of breast, thyroid, and skin cancer. It is recognized as a significant prognostic tumor suppressor in certain cancers and its tumor suppression activity was initially discovered in breast cancer. Several studies have reported differential expression of SERPINB5 during the progression from precancerous lesions to malignant lesions.^[42]^ Considering the absence of markers for early diagnosis of SKCM, SERPINB5 may aid in the early diagnosis of SKCM. Nevertheless, the role of this gene in promoting or suppressing cancer in various malignancies remains contentious. Prognostic studies have focused on many types of cancer, and the results of these studies are contradictory.^[43]^

VM genes play crucial roles in tumor development, invasion, metastasis, and other processes, influencing tumor prognosis. To assess the prognosis of patients with SKCM, a prognosis-related VM risk score system was constructed based on 5 VM genes. Subsequently, SKCM patients were categorized into high and low groups using this scoring system. The verification results demonstrated that this scoring system possesses a certain level of accuracy in measuring and predicting prognosis. Notably, patients in the low-score group were more predominantly under the age of 50 and exhibited lower pathological and T stages, GSEA analysis revealed that the VM high-score group was primarily related to epithelial keratinization and proliferation. This finding, combined with previous research demonstrating the presence of keratinocytes in the melanoma microenvironment,^[44]^ leads us to hypothesize that these VM risk-related genes may play a role in shaping the SKCM-dependent tumor microenvironment by influencing epithelial keratosis and angiogenesis, However, further investigation is needed to elucidate the underlying mechanism. The low-VM score group, in contrast, exhibited a predominant association with immunity, suggesting an influence of these genes on the immune microenvironment of SKCM. Extensive research has focused on tumor immunity, encompassing the investigation of tumor immune microenvironment and immunotherapy. According to our findings, the level of M0 macrophage infiltration increased in the VM high-score group, while the levels of CD8T, CD4T, and M1 macrophage infiltration decreased. Tumor-associated macrophages play a crucial role in the growth of tumor cells, blood supply to tumor tissue, and remodeling of extracellular mechanisms during tumor development. Initially, researchers categorized them into M1 macrophages, which can promote inflammation and anti-fibrosis, and M2,activated by IL-4, exhibiting anti-inflammatory and fibrotic properties.^[45,46]^ Subsequently, a study on ovarian cancer identified M0 macrophages share a transcription profile resembling M2 macrophages.^[47]^ Notably, an increase in M0 macrophages results in a poor prognosis for tumors.^[48]^ Therefore, we hypothesized that VM enhances the invasion and metastasis of SKCM through the enrichment of M0 macrophages, leading to poor prognosis. The tumor microenvironment modulates T-cell activity and function by regulating cellular metabolism and signaling pathways. The aerobic glycolysis of tumor cells and promotion of angiogenesis leads to glucose or amino acid competition between T cells and tumor cells, and the consumption of these key nutrients for T cell activation (such as glucose) may promote AMPK signaling by inhibiting mTORC1 activity, thereby limiting nutrient uptake and metabolism of T cells. Our findings demonstrated a significant reduction in the number of T cells within the high-score VM group, aligning with the observed regulatory process. The GSEA results also indicated that in the high-score group, significant enrichment of genes involved in the glycan degradation pathway within the high-score group, indicating that VM formation and the above results may have a synergistic effect.^[49,50]^ VTCN1 encodes B7-H4, a member of the B7 immune checkpoint molecule family. It is believed that B- and T-lymphocyte attenuators can indirectly interact with B7-H4 receptors. Studies have demonstrated that recombinant anti-B7-H4 antibodies represent a potential approach to enhance anti-tumor immunity. Neoadjuvant chemotherapy has been observed to increase the levels of CD4 + and CD8 + T-cell while reducing the B7-H4 expression levels.^[51]^ Therefore, targeting B7-H4 could offer a novel approach for SKCM immunotherapy. Targeted therapies have emerged as a significant area of interest in cancer treatment. Since a hypoxic environment promotes the formation of VM when the endothelium-dependent angiogenic pathway is inhibited, VM is considered to be one of the risk factors contributing to the resistance of tumor anti-angiogenic drugs. For example, bevacizumab induces autophagy to activate the PI3K-AKT pathway in gliomas. Additionally, stem cells can be triggered by KDR/VEGFR-2 to differentiate into endothelial cell-like phenotypes, contributing to VM formation. In this study, the sensitivity to erlotinib, imatinib, lapatinib, and sorafenib was higher in patients with high scores, suggesting the potential of targeted drug combination therapy based on these findings. Finally, we demonstrated that the prediction of 1,3, and 5-year SKCM prognosis can be visualized using a nomogram for clinical use. The ROC curve results showed the potential clinical application of the clinical prognostic model established in this study.

As an initial exploration of VM genes in SKCM, this study possesses certain limitations. The mediation mechanism of VM-related genes on the occurrence, development, and invasion of SKCM, metastasis process, tumor immune microenvironment, and the selection of sensitive drugs needs to be further studied in basic experiments, and the results of the analysis also need to be verified and supported by larger sample sizes and multi-center clinical data.

## 5. Conclusions

We have identified 5 VM genes associated with prognosis of SKCM. Additionally, we have established a novel SKCM prognosis model and validated its reliability. The findings from this study hold promise for enhancing clinical diagnosis and treatment strategies in SKCM.

## Acknowledgments

We are grateful to Wu Guangzhen of the First Affiliated Hospital of Dalian Medical University, an expert in bioinformatics statistical analysis, for his guidance in the statistical analysis of this paper.

## Author contributions

**Conceptualization:** Yubo Wang, Junwei Zong, Shouyu Wang, Ming Lu.

**Data curation:** Yubo Wang, Linxuan Zou, Lei Meng, Junwei Zong.

**Formal analysis:** Yubo Wang, Linxuan Zou, Lei Meng, Mingzhi Song, Zhuqiang Jia, Lin Zhao, Xin Han, Ming Lu.

**Funding acquisition:** Ming Lu.

**Methodology:** Yubo Wang.

**Project administration:** Yubo Wang, Shouyu Wang, Ming Lu.

**Resources:** Junwei Zong, Shouyu Wang, Ming Lu.

**Software:** Yubo Wang, Linxuan Zou, Lei Meng, Mingzhi Song, Zhuqiang Jia, Lin Zhao, Xin Han, Ming Lu.

**Supervision:** Yubo Wang, Junwei Zong, Shouyu Wang.

**Validation:** Yubo Wang, Xin Han, Junwei Zong, Shouyu Wang.

**Visualization:** Yubo Wang, Mingzhi Song, Zhuqiang Jia, Lin Zhao, Ming Lu.

**Writing – original draft:** Yubo Wang.

**Writing – review & editing:** Yubo Wang.

## Supplementary Material


















