# Supplementary material for: Establishment of skin cutaneous melanoma prognosis model based on vascular mimicry risk score
Source: Medicine (Baltimore). 2024 Feb 16;103(7):e36679. doi: 10.1097/MD.0000000000036679 (PMC10869071; doi:10.1097/MD.0000000000036679)
Supplement: Supplementary file 1 [file medi-103-e36679-s001.pdf]

Gene expression matrix after GTEX and TCGA merger

geneNames	GTEX-P4PQ-	GTEX-ZF29-	GTEX-TSE9-	GTEX-12WSI	GTEX-OOBK-	GTEX-X585-	GTEX-QCQG-	GTEX-P4QT-	GTEX-ZT9X-	GTEX-11DX	GTEX-1107	GTEX-1301	GTEX-Y8LW-	GTEX-13PVI	GTEX-T6MO-	GTEX-ZVZP-	GTEX-U4B1-	GTEX-ZVZO-	GTEX-13FT
PSMA2P3	0	0	0	0	0	0	0	0	0	0	0	0	0	0	0	0	0	0	0
OR10V3P	2.6974563	1.1755737	0	0	2.4468102	5.4758519	0	3.0690388	4.3359407	4.0258602	0	1.8334028	2.6344982	2.4991504	2.6121211	0	2.729182	3.341023	0
IGHV7-34-1	0	0	0	0	0	0	0	0	0	0	0	0	0	0	0	0	0	0	0
CPHL1P	3.1467584	0	2.1853712	1.1795725	1.1363559	0	0	0	1.6089772	0	0	0	2.8033821	0	0	2.7190989	3.7522004	2.1041076	0
TCOF1	11.309905	11.170049	11.856831	11.249748	11.885613	11.116032	10.435709	11.328476	10.767076	10.996746	11.088472	11.364711	10.990191	11.731129	11.415685	11.283052	11.289175	11.877576	11.132354
NSRP1	10.757604	10.704125	11.222683	10.355039	10.370292	11.056118	10.522257	10.96743	11.035565	11.124506	10.064196	11.15684	10.470346	10.398854	10.209886	10.583332	10.576876	10.537899	10.539792
SPPL3	12.434023	13.075685	12.683771	12.553786	12.447794	10.886139	13.272684	10.863691	10.806684	10.65202	12.815733	12.38957	12.26706	12.863085	12.749198	12.787976	11.498554	13.003248	13.283106
OPA3	9.1984346	10.034732	8.6975705	10.206235	9.4691913	9.7896116	9.5649858	10.359034	10.060078	9.9372312	8.4616498	8.6513036	9.1919742	10.358095	9.6009554	9.7275151	9.3123107	10.182013	9.5793876
OPA1	10.787825	11.290246	11.29574	11.277153	11.079739	11.490376	11.586716	11.763395	11.730635	12.050309	11.246842	11.788379	10.698583	10.868201	11.084128	11.156434	11.220468	11.035356	11.270645
AL359853.1	0	0	0	0	0	0	0	0	0	0	0	0	0	0	0	0	0	0	0
RNU6-1209P	0	0	0	0	0	0	0	0	0	0	0	0	0	0	0	0	0	0	0
RNU4ATAC18	0	0	0	1.1795725	0	0	0	0	0	0	0	0	0	0	0	0	0	0	0
AC092652.1	0	2.8750451	2.6383059	0	0	0	0	0	0	0	1.4839769	1.127913	2.4382212	2.4991504	2.3444027	0	0	0	1.8214233
RNF219-AS1	0	2.0886865	3.1471813	2.2182638	4.1587419	0	1.9690166	3.7641363	0	5.1646412	3.6958124	0	1.816841	2.9017295	3.564076	4.3974335	0	2.2951801	2.6742788
ITGA8	9.9136222	7.9206197	6.1682432	9.792934	10.011091	7.2364832	8.9268822	5.3807467	7.9120927	4.3209383	6.558263	8.3439296	9.5927442	9.080215	9.5021098	7.3062	9.1951873	7.4356557	5.3065938
ITGA9	9.3862336	8.2938531	8.1802593	9.3275549	9.2649408	5.6856465	10.084269	4.5834063	6.0706856	5.1480118	9.0920472	10.029052	9.4177378	8.6206938	9.1298495	8.1140695	9.7235766	8.3937791	7.8845869
ITGA1	9.5849548	7.9871117	9.6840285	9.0638968	10.499713	12.711063	9.7436152	11.959885	12.827479	12.741713	9.8888838	11.2462	10.114132	9.4875462	10.030626	8.7205486	11.057307	8.5363832	8.6126992
ITGA2	9.7446999	10.636671	11.061473	10.972505	9.41411	8.929649	10.500092	10.477441	8.7314308	9.7048393	10.435131	10.982681	9.4613455	9.4145769	9.9925312	10.586953	9.853344	10.460784	10.939748
ITGA3	11.862909	12.097548	12.071517	12.342009	12.285335	10.493442	11.64366	12.603849	10.335412	12.032101	12.504034	12.718298	11.471156	12.247588	12.18166	12.64424	11.582785	13.015302	12.733664
ITGA4	4.8038127	5.2615227	6.933576	6.1102276	5.1912631	10.830431	5.5933364	12.084829	11.279676	11.037971	5.1750922	5.2728074	5.8499327	4.6173838	5.5751012	4.8621562	5.5285398	5.0150603	4.9955804
ITGA5	13.850668	11.493099	12.507924	11.628079	14.285601	13.089232	12.929589	14.528546	13.786316	14.299409	12.372799	13.206276	13.717993	12.620307	12.953546	11.638218	14.272501	11.50506	9.9180218
ITGA6	11.852344	12.278279	12.573252	12.194623	12.028587	9.5742446	12.197859	9.5481687	8.0084251	7.2614355	11.686637	12.785058	11.576134	12.095109	12.433601	12.15087	12.947484	12.656152	12.767768
ITGA7	12.373574	9.6881223	10.173388	10.906334	12.748109	8.7877977	11.171686	9.0879626	9.0778887	8.0575262	11.540398	11.832386	11.310522	11.220874	11.127905	10.098583	11.965602	10.084859	10.48491
TRHR	0	1.1755737	0	1.1795725	1.7802862	0	2.3516473	0	1.6089772	3.6167598	0	0	0	0	0	0	0	0	0
AC010468.3	0	0	0	0	0	0	0	0	0	0	0	0	0	0	0	0	0	0	0
AC010468.2	0	0	0	0	0	0	0	0	0	0	0	0	0	0	0	0	0	0	0
AC010468.1	3.6487758	3.0067164	2.8149393	0	2.0080151	2.8231338	2.8310357	3.3498812	5.1900188	3.3870728	2.701596	2.7067258	2.072532	2.6749431	2.9233754	4.2273937	1.5535662	4.0061008	3.0958349
CA15P1	0	0	0	1.9627237	1.1363559	0	0	0	0	0	0	0	0	0	0	1.28151	0	1.3766709	0
CA15P2	0	0	0	0	0	0	0	0	0	0	0	0	0	0	0	0	0	0	0
UFSP1	4.3591231	4.7458162	1.4848249	4.5068527	5.10859	6.8704097	4.3304567	7.0403827	6.6596355	6.762833	2.7652995	4.5184482	3.6398427	3.7210628	2.0467176	4.4464847	3.5354567	4.9449192	4.2322168
UFSP2	9.9318925	9.8145387	9.9303013	9.4759164	9.5507808	9.6949736	10.165095	9.8944935	9.650343	9.9226547	9.72541	9.7396418	9.8122693	10.036099	9.7392338	9.819304	9.4647043	9.7751532	10.150134
C9orf40	7.0748922	7.0874508	7.1236033	6.1515988	6.6654546	8.4544815	4.8774988	8.8246108	8.2904468	8.8320578	6.3254176	6.6561668	6.1251171	6.9313801	6.9754018	6.8550122	7.0825107	6.9367633	6.6605466
[illegible]	[illegible]	[illegible]	[illegible]	[illegible]	[illegible]	[illegible]	[illegible]	[illegible]	[illegible]	[illegible]	[illegible]	[illegible]	[illegible]	[illegible]	[illegible]	[illegible]	[illegible]	[illegible]	[illegible]
