# Supplementary material for: Establishment of skin cutaneous melanoma prognosis model based on vascular mimicry risk score
Source: Medicine (Baltimore). 2024 Feb 16;103(7):e36679. doi: 10.1097/MD.0000000000036679 (PMC10869071; doi:10.1097/MD.0000000000036679)
Supplement: Supplementary file 8 [file medi-103-e36679-s008.pdf]

geneNames	GSM476606	GSM476607	GSM476608	GSM476609	GSM476610	GSM476611	GSM476612	GSM476613	GSM476614	GSM476615	GSM476616	GSM476617	GSM476618	GSM476619	GSM476620	GSM476621	GSM476622	GSM476623	GSM476624	GSM476625	GSM476626	GSM476627
A1BG	284.62033	265.5715	89.64986	125.52642	632.0727	162.20483	110.45832	324.64975	438.25864	140.71156	149.59642	271.87772	114.24474	153.1467	123.65715	80.05459	79.936584	95.37435	285.8061	364.95807	188.53568	144.42773
A1BG-AS1	44.364906	55.535072	8.276034	7.509827	51.618767	14.659687	11.50694	33.359253	61.41772	9.958149	25.151886	11.475254	8.71424	17.196125	9.754072	22.778368	11.38163	13.439475	23.610266	71.486046	24.1095	9.588967
A1CF	66.128314	67.34614	176.39042	86.683553	74.808137	165.68022	96.792517	158.94282	193.18304	153.7811	171.06872	133.28452	182.91921	221.96539	133.35663	168.91351	179.89426	125.05423	130.26318	92.227751	205.54279	260.18665
A2M	2669.9238	1499.3054	1936.6274	1026.554	1878.9744	499.52095	3207.3282	1901.5993	2327.5182	2810.2302	1450.3499	1826.4596	1040.0391	1490.0901	272.76079	1345.615	2779.0987	1892.5236	719.28824	2244.0998	735.57029	650.55199
A2M-AS1	375.55487	683.0333	162.61977	83.354095	187.67532	74.50905	190.85573	147.5098	115.11562	126.14908	206.23366	202.05676	46.83934	153.3198	63.84897	201.22002	139.98848	167.88918	85.27347	147.73108	77.536964	65.25016
A2ML1	47.065718	28.065016	38.029575	34.356565	54.284779	41.717924	38.480425	43.706764	44.113708	42.110907	41.009239	34.29523	37.017075	44.731885	39.03031	588.72732	45.333415	37.852666	38.958574	33.466349	65.831681	52.878507
A2MP1	74.60877	60.3628	198.5713	46.434578	59.045856	259.25198	164.9491	263.62262	111.16804	278.23242	215.2656	110.17364	92.26248	222.79109	205.51263	218.02562	290.48196	208.18515	213.76083	58.88882	166.96828	191.11012
A4GALT	72.91224	95.50058	105.54868	23.999374	85.63858	116.15696	108.69019	97.44289	80.82825	106.8262	102.52885	111.03311	83.426926	75.85226	99.9839	83.79692	97.53553	129.37172	92.821075	86.78678	27.95271	34.426926
A4GNT	50.042988	21.466711	35.918137	38.3325	30.675112	31.48714	25.693888	29.337336	21.014645	25.736403	38.920258	29.262764	25.695215	30.506054	31.412905	42.93711	34.74062	33.199932	30.715366	36.71921	79.55102	92.89344
AA06	76.10415	84.16065	73.78246	63.669006	105.43011	87.17882	91.17937	69.12861	74.867676	72.203514	76.87715	68.98726	78.02133	86.100876	78.585	91.65371	84.26575	62.937725	78.211006	74.55936	108.0079	100.61787
AAAS	294.50403	282.194	137.05103	38.610443	275.03366	144.05583	161.41266	178.1377	120.36361	168.0143	175.30931	184.99687	182.19473	141.90234	163.86087	130.4719	154.07214	155.10147	194.36456	264.58792	131.85374	161.57755
AACS	346.7058	384.44522	325.75204	54.082367	397.30588	77.76613	224.74838	191.0561	267.9001	87.438576	151.4506	137.69826	215.27911	213.97197	261.7887	497.8619	151.05745	103.77933	215.6718	305.74594	278.85263	194.93387
AACSP1	9.667528	30.612553	16.664108	6.6459045	2.4051332	19.446651	11.945131	15.872257	7.0415726	8.343125	8.129975	11.577722	15.281548	16.363047	21.934277	23.8178	10.029566	15.477195	12.730105	6.9039373	10.663901	2.7508416
AADAC	13.722479	32.12047	23.052803	3.9941938	6.740212	27.30577	23.069893	21.499338	40.463505	28.758324	27.697977	38.431877	25.343922	27.95545	24.920033	57.982697	19.279007	18.796434	33.161163	24.321772	62.741886	39.360397
AADACL2	3.331676	36.55108	47.20484	22.796387	45.09399	33.63798	42.231983	39.541218	66.4366	53.14994	46.035374	40.615734	46.816025	68.145966	45.91251	3369.4478	42.565495	65.77274	48.32941	38.027004	129.49649	11.547518
AADACP1	17.395176	52.59142	22.184324	1.0330063	3.1262355	32.128223	26.284842	29.876244	28.365705	30.519358	21.88906	32.60338	18.54858	22.97447	24.67866	16.507996	26.374546	39.342827	17.178463	31.030983	13.382227	10.792922
AADAT	148.43077	265.90076	22.041847	11.498204	79.873604	69.587006	184.54192	17.708143	27.59379	23.74336	27.741938	31.798634	142.52403	39.18026	23.416454	90.56602	56.28244	15.585727	45.36242	22.607733	148.96628	152.82632
AAED1	524.7687	626.31024	193.58719	106.45831	529.2367	528.62384	175.46751	162.81746	312.5469	85.650696	222.42726	112.15293	566.40967	122.95789	147.56458	617.4517	286.89935	144.45674	143.95712	446.84723	163.99112	137.95125
AAGAB	221.30861	353.76249	93.920119	91.463163	272.83209	117.68665	119.55062	141.44428	223.3286	94.318173	97.813258	82.148996	257.08299	88.404027	76.394192	102.3539	98.501587	119.38171	101.46692	266.29419	115.10391	115.24744
AAK1	279.48194	242.58039	231.50148	198.89043	239.32046	181.80739	241.85094	215.78371	192.53	107.15091	230.38582	72.200386	206.14938	183.72928	81.810905	159.3729	210.18699	86.890769	132.0898	208.24952	145.22315	140.39236
AAMDC	659.1344	256.96034	134.68414	98.41248	413.28134	188.38917	104.96878	184.3175	263.4613	156.59696	124.43635	167.32938	235.02026	190.07245	179.77172	181.27534	166.25368	144.66712	133.17113	385.41892	272.09498	253.49424
AAMP	545.7014	659.1743	220.25674	176.77863	773.7432	558.1708	256.5961	352.91678	249.85211	281.59592	283.4052	259.32922	406.85104	199.14601	612.76306	258.5991	286.23947	179.5963	524.6707	885.60724	331.2722	330.71045
AANAT	18.488317	33.134922	47.542416	5.73996	35.293118	46.466278	46.23996	34.8609	38.18397	51.042725	43.11357	38.673676	39.76331	37.85035	50.431385	39.09164	41.285572	61.434326	35.643314	15.512684	17.652834	15.398734
AAR2	515.8633	582.09106	330.6736	347.82553	608.7438	443.79343	367.36588	416.36465	551.0851	317.02698	380.9833	367.71246	341.8836	319.3721	738.04296	420.8547	329.1744	516.19244	567.68634	524.6489	587.81683	547.03046
AARS	1432.8939	1155.1354	1021.8094	519.62573	1204.5381	755.8192	767.73865	1573.6215	1175.0156	1222.1709	724.9087	892.2594	1989.2572	988.6296	261.2267	481.25323	1171.2286	492.0794	1053.9261	1438.9237	1157.7268	907.4952
AARS2	159.46736	125.75035	128.16038	41.127881	142.12481	137.36016	144.37316	146.19693	134.0908	184.92931	149.85478	188.11419	158.36916	129.03404	231.99291	133.28376	144.73832	203.30207	197.92585	180.3824	118.95784	167.00507
AASDH	218.05692	200.10051	126.28678	125.14234	205.97003	123.47594	140.36898	159.09831	157.01397	170.93995	195.38154	118.66166	120.29325	121.22262	211.1498	195.20217	117.67885	115.70782	173.71863	295.70647	189.69219	181.08514
AASDHPPT	749.34556	1063.4734	451.71409	259.46565	798.72333	622.41238	1108.258	365.74364	529.84162	801.45732	546.90806	430.03956	1010.2799	437.6362	798.86139	541.47	525.78634	618.76771	883.37756	692.30497	584.0175	646.0436
AASS	195.77729	158.05139	116.63262	17.83041	102.69537	75.147983	90.45786	91.660413	89.745705	96.23595	77.914173	116.15611	143.95348	147.11154	132.65455	83.991881	66.993244	88.042783	59.393807	149.35174	289.93252	233.8865
AATF	1587.6439	1322.2692	670.0243	541.1931	1134.1436	917.18774	702.25336	488.6312	531.6391	795.09015	736.73883	621.3102	1233.2058	659.8954	625.6118	557.9574	724.21643	692.3595	861.1915	1471.6965	633.54156	598.4504
AATK	64.04781	153.17352	146.50397	16.553577	277.58612	35.43197	703.7309	54.32982	102.50595	205.195	36.641743	245.01535	61.820404	95.395	64.79576	81.02625	39.610043	301.2173	379.23068	277.301	204.58968	128.38312
AB488780	152.92091	130.32632	86.93575	18.416157	74.050095	77.1247	83.50473	59.598507	40.896152	87.84076	112.07222	59.68995	111.45913	63.62875	114.23708	60.505802	90.786934	74.242035	62.39648	122.07513	112.26894	133.30139
ABAT	260.9123	211.50218	143.64407	124.31878	192.20977	146.0476	61.124177	31.306998	49.679499	46.772976	120.25921	45.406799	54.483305	67.342761	112.90437	148.53966	90.202735	23.715105	29.776753	185.8117	121.52504	90.931061
ABCA1	471.54594	530.62215	297.69161	328.68844	467.63209	439.09806	592.4167	234.30827	180.72375	335.17784	544.47678	109.93754	352.67079	196.84322	112.34108	376.16676	469.20424	434.22436	204.84183	387.73869	318.49684	207.71437
ABCA11P	90.203156	77.26125	117.49474	67.15465	108.39188	95.05152	85.35021	89.563545	110.83109	102.90979	122.61149	89.02987	125.23093	89.56169	132.65968	108.06267	125.80206	114.16663	110.90508	84.2055	449.89722	479.07962
ABCA12	55.849514	51.646217	108.83205	34.60106	74.97384	106.54192	100.04981	104.81794	104.14433	130.87956	102.29988	133.81157	82.29206	96.200035	105.68263	448.03375	97.81544	144.43048	108.99252	92.12378	48.459854	56.38973
ABCA13	36.384043	37.385298	33.058431	8.1077917	12.291019	29.802087	20.703067	42.907668	30.591557	51.160662	30.322744	23.79409	25.243524	31.549398	32.908615	28.91364	36.761046	59.705188	31.754786	10.687844	31.636632	30.399483
ABCA17P	64.091194	54.487057	67.58364	7.454141	59.921722	40.807217	25.941572	68.55653	77.572105	88.022026	40.16186	36.06537	38.503265	83.03577	33.453037	18.371893	27.958452	30.040735	34.178066	88.746895	69.28536	65.24245
ABCA2	157.32757	134.78657	110.62265	26.177117	110.40896	93.120843	170.35534	100.6657	92.185737	93.164631	134.76133	125.21057	85.125365	98.736791	69.32881	109.73508	105.54627	92.003073	79.017869	98.863365	105.64919	71.766403
ABCA3	461.40833	280.11475	299.9655	142.95697	197.1419	22.344553	75.82175	180.16165	354.14053	50.493248	153.91928	108.24571	104.5713	156.32053	11.379562	67.91594	19.821133	55.621807	28.609612	193.52074	33.805817	9.657604
ABCA4	6.8009311	34.00427	54.913589	9.2008135	39.812326	52.786536	52.127024	54.715143	37.152352	52.765083	58.199708	61.742334	42.359968	44.38939	52.142642	34.501986	59.672229	69.371136	48.843747	23.131236	16.624535	11.494254
ABCA5	138.48771	291.69736	86.950325	84.417477	497.73566	45.321736	66.459995	257.86531	362.09144	203.75899	136.66907	194.54336	86.968069	178.98357	129.09105	158.52374	88.348522	140.5444	276.25499	249.32325	469.19015	285.29771
ABCA6	153.36359	47.61675	79.72293	84.58131	19.926064	30.8166	60.446247	34.04188	43.48415	29.979435	98.37168	42.551525	33.614075	47.806946	23.129484	133.45523	99.90426	35.302902	33.08579	50.20894	35.431496	31.35292
ABCA7	100.38241	99.10059	107.30854	42.932606	127.346	92.6148	117.90619	94.821526	91.62297	108.68527	119.49669	113.36565	103.16458	83.00631	71.97577	75.416306	107.42081	125.54592	96.76112	118.13435	72.73637	67.58653
ABCA8	587.8296	220.34288	81.702605	51.081087	36.126267	28.601119	75.375389	29.438042	43.519925	46.909816	154.17855	30.470229	77.962599	50.064535	71.93278	109.28439	52.346309	42.030463	114.22922	85.901904	220.17748	149.04944
ABCA9	64.309227	17.898714	49.294147	22.050765	37.591692	32.963162	40.961631	37.026352	30.357949	55.326005	48.336332	35.943034	25.954479	46.754845	38.249416	61.289878	50.926542	51.196514	37.86941	54.464937	97.427081	72.696239
ABCB1	63.702555	172.21189	74.242375	63.517525	22.878583	58.889263	73.263785	59.283167	47.840163	52.899427	164.06921	41.075352	100.73847	55.931286	55.392054	59.323071	77.194955	63.271492	77.226648	101.32553	58.888347	71.696178
ABCB10	628.7191	656.84625	222.62724	346.8039	740.1498	289.18497	167.00935	596.2845	894.151	177.68732	376.58936	174.14099	509.75055	383.44272	216.52423	441.05212	353.44885	131.49113	273.17386	545.5227	599.3406	499.9536
ABCB11	8.3457304	8.6892175	23.479051	10.666932	12.809618	24.320474	22.626309	24.723227	19.114591	22.215678	21.129929	25.477979	24.594346	21.155355	23.144054	21.642464	23.595047	31.43461	23.109174	6.9279047	7.2021205	11.070533
ABCB4	79.972895	146.91411	199.01453	67.624242	141.97522	33.028918	194.48718	34.773357	26.039991	46.007873	172.51671	48.011526	62.310334	60.281855	28.96125	54.494815	45.553489	35.918004	87.812265	433.55016	80.899547	64.21999
ABCB5	19.344622	169.86386	36.784918	24.875376	2213.5252	40.035797	46.879442	641.37567	760.90763	466.13047	42.545522	370.39646	79.313313	1280.1638	34.314387	49.073221	36.597652	545.62375	350.49178	1011.4406	286.91466	276.59703
ABCB6	120.42638	153.11228	104.81937	29.078413	272.47282	119.58956	175.48129	120.07786	116.93711	104.30568	84.443905	120.59214	102.01239	143.43729	114.76865	118.98192	91.013763	115.92579	151.46961	154.91563	113.11752	99.056318
ABCB7	418.93982	403.1951	256.2601	212.52025	461.69244	270.9343	248.19014	163.42387	228.79388	180.76343	267.1608	208.34508	451.08835	182.726	347.957	259.8757	290.26428	180.5163	193.66417	422.34122	217.08578	233.0616
ABCB8	154.36472	232.0156	168.90455	59.208891	413.17785	148.7769	165.59733	162.00244	150.39536	160.34413	163.68103	203.91325	146.19365	152.12088	163.50024	146.78379	119.19598	188.94832	167.64685	261.39315	166.80635	127.79865
ABCB9	202.80937	271.22487	350.51367	73.450729	119.53989	290.05238	292.01998	253.106	175.06079	286.20605	255.1922	171.24299	123.60417	359.37854	279.19088	228.21377	293.37158	262.0399	289.50344	115.46483	181.14295	183.93541
ABCC1	408.37619	547.06442	189.53681	244.84183	323.73078	266.05312	83.89883	207.36546	204.61847	112.62759	362.07278	80.825397	457.98983	154.25781	15.810111	330.02046	237.20121	39.296986	191.35574	553.79957	361.92962	298.85342
ABCC10	380.474	262.28225	197.33767	135.95653	491.89929	197.7724	199.99508	435.01398	494.1379	242.10231	215.29852	177.41691	550.9144	290.12987	262.37139	196.548	321.0637	207.18018	209.1302	482.04172	229.3879	300.09895
ABCC11	11.411599	9.345902	49.594361	7.1438185	3.68728	50.229735	43.934257	49.059217	39.155143	50.926525	43.46672	55.34136	38.840544	40.702309	45.338544	34.452099	51.105625	55.829304	51.263022	33.158804	20.424451	20.066474
ABCC12	6.1689708	9.9143975	26.602406	3.211769	15.535736	20.944982	30.715154	26.35587	28.001424	29.405265	26.721834	26.528086	27.025514	22.686103	32.11416	22.291439	23.222488	32.769831	27.701011	16.560798	13.535923	12.323166
ABCC13	21.807201	21.819684	47.829225	19.978441	22.690789	47.475052	35.708149	47.011293	40.117047	53.933896	44.266802	41.580826	37.218201	54.462037	45.69952	40.198767	46.511806	53.68946	42.947747	26.60022	33.806509	36.893928
ABCC2	22.67851	22.951342	12.603319	9.827579	50.839478	13.211464	4.8200665	108.46945	101.16277	72.03111	18.676565	35.551468	23.29899	92.70298	14.547943	21.76244	18.800833	39.141518	135.22948	186.36792	33.066727	86.4452
ABCC3	80.512483	56.230817	163.42278	82.882149	71.375014	90.920091	157.46021	81.422692	65.576675	93.327549	141.40205	88.584871	76.651835	73.569904	76.719317	118.19392	97.855699	100.72255	76.939893	88.529962	47.273575	34.842767
ABCC4	135.21601	136.30751	76.899172	87.249958	122.96708	222.58682	119.88835	77.662728	69.264181	51.946954	99.920049	118.73062	139.72512	110.64469	44.436376	103.55949	122.29587	68.144416	52.808129	203.76134	141.91866	95.193063
ABCC5	309.9306	237.49554	121.21905	57.311606	390.34587	128.19658	274.41712	295.00564	331.54784	129.04246	116.61417	134.88062	187.5275	133.96164	108.80708	131.18745	153.12729	141.57667	155.65165	303.4426	232.84375	262.51961
ABCC6P1	24.896173	8.607482	17.984026	23.832077	10.794261	23.189697	9.844826	17.593096	20.10701	15.627839	26.146008	25.799343	23.11635	24.277916	20.563408	27.096088	28.328781	28.925539	29.61682	12.965096	42.121704	36.47042
ABCC8	6.381156	7.9196779	28.992062	8.7823082	14.902105	31.222513	29.183558	27.546815	23.718284	30.337025	26.635368	30.128948	21.295189	32.138714	26.604996	28.657457	28.370814	24.848922	24.213483	19.939636	17.841611	22.043496
ABCC9	42.568974	53.516889	45.928396	19.033062	27.030307	57.942211	45.650376	48.351769	48.653264	53.03311	75.128134	39.87531	98.522972	51.755504	35.306669	60.487982	85.424337	70.851386	44.149818	35.95322	55.816844	51.403463
ABCD1	190.44476	182.18288	712.3968	79.180626	947.61035	334.26987	197.5351	745.8939	311.9741	346.65826	164.37277	465.2709	175.02599	799.62256	110.50759	117.08952	202.08034	245.81082	902.44257	364.88712	213.60303	248.34753
ABCD2	17.322699	8.477416	34.830894	12.793363	2.9825938	37.66908	22.764353	31.469843	35.33087	40.361465	61.26776	25.466896	25.606318	35.391975	28.333593	21.405367	36.9368	34.680573	28.859768	27.612625	30.325487	22.066782
ABCD3	633.8023	683.30403	445.03012	157.39429	792.4058	357.74653	456.61345	687.64989	792.17403	822.16566	368.71466	335.01761	521.18709	461.32395	815.36886	719.05538	568.12662	582.046	777.07652	1147.2067	651.31825	848.80599
ABCD4	214.04428	173.93111	97.192312	79.489303	122.73946	79.159265	94.49308	93.44733	157.53909	76.299448	189.81679	73.511947	159.39964	63.109844	79.875236	154.34628	121.16246	127.82647	148.18943	122.88891	153.29163	161.15523
ABCE1	1157.6761	1655.9667	425.18386	540.53625	1466.2995	883.71223	929.04705	1036.5796	1195.0803	633.6416	574.64661	398.85267	1449.1501	847.2148	695.5469	551.50388	644.79277	410.86438	1132.8699	1244.5367	896.0252	930.38653
ABCF1	943.5865	785.92065	620.36786	354.1004	816.104	1230.0917	704.07117	984.294	933.0361	1252.4478	787.6863	444.74176	1541.115	615.34045	1581.0967	573.0068	1010.2481	1528.2094	1693.198	996.7325	688.0157	822.61646
ABCF2	405.89961	421.6904	229.5523	72.356778	523.79418	195.94846	303.05171	249.91323	234.20903	260.97119	179.13714	195.41724	228.93681	183.54674	182.05406	183.05147	163.34258	154.66222	234.92172	758.22903	261.31731	239.53162
ABCF3	327.29535	274.8967	192.90157	89.65105	346.8665	199.60435	398.48862	261.1347	229.35312	138.28047	185.08522	141.41362	226.74194	178.2841	232.40588	155.10544	162.46815	125.62766	207.78186	350.25168	209.14929	203.75084
ABCG1	270.78656	250.14982	172.9421	208.45704	510.6589	192.37746	107.60578	92.241959	94.41722	130.89048	337.69572	73.333265	114.1006	101.81368	61.138308	266.99532	241.07531	145.98923	114.2328	200.58421	72.892755	71.789328
ABCG2	59.550755	108.00489	89.00955	43.892017	74.10942	51.461555	85.70909	147.5556	94.43279	73.32498	112.49552	59.5365	83.35699	74.36791	82.82323	86.17827	78.566925	64.84585	69.05987	187.96187	82.138374	71.009155
ABCG4	52.369026	57.63248	138.26532	11.018008	74.69563	162.19527	152.51135	131.65154	109.08292	175.461	119.04727	116.47807	100.51907	110.68393	127.97688	117.72338	144.84346	178.44551	129.68892	67.59503	28.956196	19.028162
ABCG5	31.474543	13.742344	85.420654	8.328843	35.09904	87.84697	52.45991	77.86265	59.972767	78.85039	114.00053	158.13954	88.39786	60.45724	68.34611	59.862503	93.65895	67.21401	57.00221	22.817245	42.2527	71.10337
ABCG8	4.0804915	20.666164	22.257208	15.275368	2.5218027	21.235548	27.780388	23.9678	14.47751	21.120714	20.572819	19.195942	27.833849	19.302303	16.329958	13.922298	20.329086	28.1244	24.325594	4.283805	3.276536	1.5300622
ABHD1	42.516877	36.122142	61.01083	49.651857	54.153879	53.227797	48.254039	46.971783	55.815927	62.808513	59.91004	56.634324	58.534393	72.796668	54.520491	65.083022	55.902818	51.157818	54.825666	37.414586	98.48089	96.934572
ABHD10	610.17426	1298.2629	269.56543	407.65122	1146.7808	279.06795	706.24229	340.63265	470.85824	229.00461	478.52937	306.25954	517.64402	391.96263	318.16985	306.51893	306.16426	216.67812	385.72368	962.3116	521.21972	490.50248
ABHD11	151.07021	145.75722	197.62405	68.143194	226.65556	127.60175	239.02028	184.91826	149.13316	182.80478	149.26679	181.25991	126.21302	177.69095	125.11498	135.64275	145.0535	177.33496	154.60924	392.81196	154.1022	153.39951
ABHD12	587.21745	573.54638	382.76161	137.34683	1020.7154	479.89916	445.74445	295.48118	261.42206	305.51096	176.9549	457.42602	225.91748	224.28455	354.72631	271.48554	403.0258	392.31922	276.35717	941.76245	328.90327	355.75397
ABHD13	353.39416	394.77072	71.588323	97.816354	106.1574	61.497838	42.77278	61.972332	111.13411	65.232391	152.24261	200.61316	71.12751	78.341965	108.8823	129.60987	71.805744	55.911017	72.322675	207.78231	143.07234	199.02853
ABHD14B	752.2502	430.38684	851.60596	341.7708	1025.9066	880.40356	388.95746	1095.1486	832.21246	1044.9769	1089.274	1718.9177	1386.9364	1156.4755	950.9088	763.22144	394.66046	1456.8082	1142.0284	834.68176	479.38306	400.1388
ABHD15	274.31723	152.62733	158.08595	147.46707	232.67606	64.5217	138.88583	78.77067	142.09738	118.29303	327.09335	75.5757	133.4495	176.20436	11.336846	184.19878	141.49583	48.794483	206.81625	236.62299	150.1828	81.22178
ABHD16A	458.4428	375.17428	215.75107	180.34203	436.69808	235.40156	569.19981	367.54259	433.60111	516.13765	360.7327	251.72047	416.65692	199.03311	292.69274	297.23148	378.76523	569.8584	446.06862	471.22268	173.71718	244.12412
ABHD16B	9.116473	30.699713	27.599024	5.233364	9.832861	28.044348	27.927317	30.325138	20.377207	33.434475	20.11817	35.034683	25.771732	22.561361	21.78461	16.865738	25.741884	32.46326	24.309727	6.6641536	4.12174	2.8600276
ABHD17A	1762.3126	1904.7026	1561.7611	1023.0266	2088.488	1054.9586	2135.6082	1289.2101	746.1648	1319.1437	2302.7786	2280.9534	918.53546	845.77167	2053.5752	1263.9952	1220.2562	1213.9313	913.06036	2153.6418	1249.6494	1094.235
[illegible]																						
